# Four-Dimensional Flow MRI for Cardiovascular Evaluation (4DCarE): A Prospective Non-Inferiority Study of a Rapid Cardiac MRI Exam: Study Protocol and Pilot Analysis

**DOI:** 10.3390/diagnostics14222590

**Published:** 2024-11-18

**Authors:** Jiaxing Jason Qin, Mustafa Gok, Alireza Gholipour, Jordan LoPilato, Max Kirkby, Christopher Poole, Paul Smith, Rominder Grover, Stuart M. Grieve

**Affiliations:** 1 Imaging and Phenotyping Laboratory, Charles Perkins Centre, University of Sydney, Sydney, NSW 2006, Australia; jqin2356@uni.sydney.edu.au (J.J.Q.); mustafa@grievelab.com (M.G.);; 2 School of Health Sciences, Faculty of Medicine and Health, University of Sydney, Sydney, NSW 2006, Australia; 3 Department of Radiology, Faculty of Medicine, Aydin Adnan Menderes University, Aydin 09010, Turkey; 4 ANU Medical School, Australian National University, Canberra, ACT 2601, Australia; 5 iCoreLab, North Sydney, NSW 2060, Australia; 6 Epworth Radiology, Waurn Ponds, VIC 3216, Australia; 7 Macquarie University Hospital, Macquarie Park, NSW 2109, Australia; 8 Lumus Imaging, St George Private Hospital, Kogarah, NSW 2217, Australia

**Keywords:** cardiac magnetic resonance imaging, 4D-flow, cardiac imaging, imaging analysis, left ventricular function

## Abstract

Background: Accurate measurements of flow and ventricular volume and function are critical for clinical decision-making in cardiovascular medicine. Cardiac magnetic resonance (CMR) is the current gold standard for ventricular functional evaluation but is relatively expensive and time-consuming, thus limiting the scale of clinical applications. New volumetric acquisition techniques, such as four-dimensional flow (4D-flow) and three-dimensional volumetric cine (3D-cine) MRI, could potentially reduce acquisition time without loss in accuracy; however, this has not been formally tested on a large scale. Methods: 4DCarE (4D-flow MRI for cardiovascular evaluation) is a prospective, multi-centre study designed to test the non-inferiority of a compressed 20 min exam based on volumetric CMR compared with a conventional CMR exam (45–60 min). The compressed exam utilises 4D-flow together with a single breath-hold 3D-cine to provide a rapid, accurate quantitative assessment of the whole heart function. Outcome measures are (i) flow and chamber volume measurements and (ii) overall functional evaluation. Secondary analyses will explore clinical applications of 4D-flow-derived parameters, including wall shear stress, flow kinetic energy quantification, and vortex analysis in large-scale cohorts. A target of 1200 participants will enter the study across three sites. The analysis will be performed at a single core laboratory site. Pilot Results: We present a pilot analysis of 196 participants comparing flow measurements obtained by 4D-flow and conventional 2D phase contrast, which demonstrated moderate–good consistency in ascending aorta and main pulmonary artery flow measurements between the two techniques. Four-dimensional flow underestimated the flow compared with 2D-PC, by approximately 3 mL/beat in both vessels. Conclusions: We present the study protocol of a prospective non-inferiority study of a rapid cardiac MRI exam compared with conventional CMR. The pilot analysis supports the continuation of the study. Study Registration: This study is registered with the Australia and New Zealand Clinical Trials Registry (Registry number ACTRN12622000047796, Universal Trial Number: U1111-1270-6509, registered 17 January 2022—Retrospectively registered).

## 1. Key Messages Regarding Feasibility

(1) What uncertainties existed regarding the feasibility?

The uncertainties regarding the feasibility included the clinical practicality of the 2D-PC and 4D-flow analysis procesess, the equivalence of flow measurements obtained from 2D-PC and 4D-flow images, and the reproducibility and reliability of the standardised image analysis process.

(2) What are the key feasibility findings?

The feasibility findings indicated that the image analysis process could be practically implemented as part of a clinical process. The pilot analysis supported the equivalence between 2D-PC and 4D-flow measurements. The performance of the trained annotators was closely correlated with that of the experts, thus supporting the reproducibility and reliability of the standardised image analysis process.

(3) What are the implications of the feasibility findings for the design of the main study?

The main implication of the feasibility findings was to inform the iterative improvement of the standardised image analysis process. The practical approach to image analysis also enabled upscaling of the study. The pilot analysis provided confidence in achieving positive outcomes in the subsequent phases of the study.

## 2. Background

Accurate measurements of heart volume and function underpin clinical decision-making in cardiovascular medicine. In particular, measurements of ventricular size and volumes and ejection fraction are universally used to quantify ventricular dysfunction in the setting of right and left ventricular heart failure—the common end-points of multiple disease processes and key causes of mortality and morbidity [1]. Cardiac magnetic resonance (CMR) imaging is the current gold standard for the assessment of cardiac function [2], but it is expensive and time-consuming and requires considerable acquisition and analytical expertise to perform reliably. Transthoracic echocardiogram (TTE) is the current preferred cardiac imaging modality in most clinical environments due to its cost-effectiveness and availability. However, it has several limitations: it is largely a two-dimensional imaging modality, sensitivity and specificity are highly operator-dependent, and imaging quality and resolution vary with body habitus. Computer tomography (CT) has also been used for cardiac evaluations, but it uses ionising radiation and provides limited functional and flow information. CMR uses nonionising radiation, is reproducible, and can provide both flow and dynamic functional data at high spatial and temporal resolutions [3]. In addition to clinical needs, accurate measurements of cardiac function, volumes, and flow are essential for the validation and testing of new therapeutic options in clinical trials. Prior data comparing CMR with TTE showed that the improved interstudy reproducibility of CMR resulted in a reduction in the sample size needed to detect clinically meaningful change by approximately 50–90% [4]. A reduction in the time and labour required to acquire and process the CMR exam would reduce study costs, making broader applications in both clinical and research settings more feasible.

The key imaging data required for evaluating cardiac function are provided by the dynamic cine and flow images. Most clinical indications also require delayed gadolinium enhancement (DGE) images to visualise myocardial scarring, infiltration, or inflammation. Although rapid and/or volumetric DGE techniques exist, they are beyond the scope of this study. Other anatomical or planning sequences (e.g., MR angiograms and axial FIESTA-type images) are also routinely acquired, as well as specific myocardial characterisation sequences (e.g., perfusion and T2-weighted or T1-mapping sequences) depending on the clinical indication. There is no universal consensus regarding how these sequences are deployed, and there is wide variation in the total number of sequences deployed and time taken in any given clinical exam [5].

Cine imaging is an essential part of almost every CMR exam, particularly for heart function evaluation. These parameters permit a standardised evaluation of ventricular dynamics, morphology, and valve function but require considerable time and skill to acquire correctly [1]. Measurements of cardiac volumes are typically performed using a short-axis stack (SAX) of two-dimensional (2D) slices spanning the ventricle from base to apex. Typical acquisition times of 4–8 min for full heart SAX coverage are common, depending on the resolution and patient factors [6]. To measure cardiac volumes, SAX images are contoured manually or semi-manually to identify endo- and epicardial borders in order to compute ventricular end-diastolic volume (EDV), end-systolic volume (ESV), stroke volume (SV), ejection fraction (EF), and myocardial mass. This process can be labour-intensive and requires considerable expertise to perform reliably and accurately.

Flow information is complementary to cine imaging and is usually acquired using 2D-phase contrast imaging (2D-PC), which provides qualitative information on valve function, as well as quantitative information on flow velocity and volume, regurgitation fraction, and additional information on stroke volume. The acquisition of 2D-PC is usually performed at slice planes located in the ascending aorta (AscAo), main pulmonary artery (MPA), and, sometimes, aortic valve (AV). Each slice plane is placed perpendicular to the structure of interest during acquisition, which requires meticulous planning and considerable expertise to perform accurately and is a time-consuming process [6]. Dynamic structures such as the aortic valve also tend to move out of the plane over a cardiac cycle, resulting in suboptimal image quality. Both cine and 2D-PC imaging are acquired over several breath-holds, which can be challenging for patients with dyspnoea or elderly and paediatric patients, resulting in degraded image quality.

While 4D-flow MRI has been available for nearly two decades, the complexity of this technique has limited its incorporation into broader routine clinical use. The recent availability of high-performance gradients improved eddy-current correction methods, while the development of enhanced accelerated imaging techniques offered by commercial software packages have made 4D-flow a potentially viable tool for clinicians [7,8]. Four-dimensional flow permits isotropic volumetric image acquisition of flow information over the entire chest cavity in a single free-breathing acquisition of 5–10 min [3,7,9]. With a free-breathing exam and a reduction in the planning requirement, 4D-flow minimises user interaction to improve scan time and patient experience [10]. Similarly, 3D-cine acquires volumetric coverage of the entire heart with free breathing or over one or two breath-holds, eliminating the need for extensive planning of acquisition planes, therefore reducing scan time and improving patient experience [6]. Both 3D-cine and 4D-flow acquisitions are isotropic; therefore, the images can be reformatted or analysed in any plane or orientation post-acquisition to more accurately and reliably quantify flow and cardiac volumes and size [3,6]. A disadvantage of both 3D-cine and 4D-flow compared with conventional 2D sequences is a lower spatial resolution. However, several small-scale cohort analyses have shown no meaningful impact of the difference in resolution on flow or volumetric measures [11,12,13,14]. In addition, there is a lack of a standardised approach to the processing and analysis of 3D-cine and 4D-flow data. The reliance on highly trained specialists to perform measurements also renders the techniques costly and difficult to generalise to wider clinical use beyond specialised centres [15]. For 3D-cine and 4D-flow to be viable routine clinical techniques, there remains the need to demonstrate that the measurements obtained from these techniques are accurate for clinical evaluations and that the acquisition and analysis methodologies can be applied widely in a scalable manner and through standardised protocols and not just at highly specialised centres [8]. There remains a lack of direct, systematic data comparing the in-field accuracy of 3D and 2D imaging sequences, and there has been no substantial evaluation of these imaging methodologies and related analyses in a clinical setting.

This paper describes the study protocol of a prospective, multi-centre non-inferiority study, 4D-flow MRI for cardiovascular evaluation (4DCarE), which tests the accuracy of cardiac flow and volume measurements and overall functional evaluation of an up-front compressed 15 min functional exam (CMR_FAST_), utilising two 3D imaging techniques (3D-cine and 4D-flow MRI) compared with standard CMR acquisition using cine SAX and 2D-PC sequences (CMR_STD_). We also present the preliminary results of a pilot cohort comparing flow measurements between 2D-PC and 4D-flow to support the design and the continuation of the study. The overall primary purpose of 4DCarE is to provide evidence to support a rapid imaging acquisition technique using 3D-cine and 4D-flow for the quantification of cardiac function and to understand the strengths and limitations related to this technique.

The 4DCarE study will test the following primary hypotheses:

- Four-dimensional flow MRI is non-inferior to 2D-PC flow for the quantification of aortic and pulmonary artery flow in a routine clinical setting.
- Three-dimensional cine is non-inferior to the conventional SAX cine for cardiac volume measurements in a routine clinical setting.
- The overall functional characterisation of the whole heart using CMR_FAST_ is non-inferior to the conventional (CMR_STD_) protocol within defined clinical parameters.

For each of the hypotheses, we will seek to define the clinical conditions within which they are true (e.g., for “all comers” vs. for specific clinical subtypes).

In addition to the primary goal, we will also formally evaluate the feasibility of training non-CMR specialists (annotators) via a standardised process and using codified image contouring protocols to perform routine post-acquisition contouring for volume and flow measurements. The aim of the evaluation is to determine whether trained annotators can perform these measurements at a clinically acceptable level of accuracy and reliability compared with CMR specialists.

## 3. Methods

### 3.1. Protocol

#### 3.1.1. Study Design

This is a prospective, non-randomised, multi-centre study. Clinical data were collected from three clinical sites: Macquarie Medical Imaging (Macquarie University Hospital, NSW, Australia), Sydney Adventist Hospital (Wahroonga, NSW, Australia), and Epworth Medical Imaging (Waurn Ponds, Victoria, Australia). The data were managed via a central core laboratory (iCoreLab, North Sydney, Australia). Imaging acquisition quality control, data transfer, and clinical review at each clinical site were coordinated by the principal investigator. Following case registration and patient consent, the clinical data were transferred to iCoreLab using the iCoreRouter platform (iCR, North Sydney, Australia), anonymized, and distributed to the Imaging and Phenotyping Laboratory (IaPL) for blinded analysis. Clinical reporting was performed adhering to local reporting standards.

#### 3.1.2. Ethical Considerations

The study protocol was approved by the human research ethics committee of the National Statement on Ethical Conduct in Human Research (2007), based on the Guidelines for Good Clinical Research Practice (GCRP) in Australia. All published findings will be in an aggregated and non-identifiable format.

#### 3.1.3. Site Selection

Prior to the inclusion of each site in the study, the principal investigator of the study provided standardised training for radiographers and set up a standardised protocol. All three sites are clinical imaging sites located within tertiary hospital settings capable of performing routine CMR and 4D-flow acquisitions.

#### 3.1.4. Study Participants

**Figure 1:**
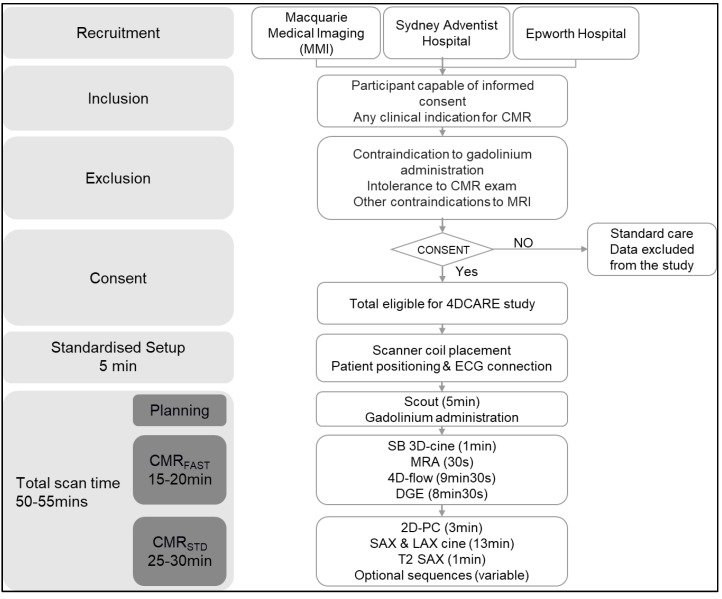
The 4DCarE study flow chart and summary of imaging protocols. CMR: cardiac magnetic resonance; CMR_FAST_: rapid CMR protocol; CMR_STD_: conventional CMR protocol; DGE: delayed gadolinium enhancement; MRA: magnetic resonance angiography; SB: single breath; SAX: short-axis stack.

The 4DCarE study recruitment began in August 2017 and is ongoing. The flow chart for the study is presented in Figure 1. The recruitment target is 1200 participants. Participants were individuals referred for a cardiac MRI for any clinical indication who were able to provide informed consent. Participant eligibility was assessed by the central study coordinator at the time of protocolisation and consent was obtained prior to imaging acquisition. Inclusion criteria for CMR were any participants referred by a health professional for CMR at the participating study sites who were capable of and willing to give informed consent. Exclusion criteria were participants with contraindications to gadolinium contrast, those who were unable to tolerate the complete exam, and any other contraindication to MRI.

#### 3.1.5. CMR Acquisition

Data were acquired on two MRI scanner types: a GE Medical Systems 3T 750W MRI (GE Healthcare, Chicago, IL, USA) at Macquarie Medical Imaging and Sydney Adventist Hospital) or a GE Medical Systems 3T Signa Architect (GE Healthcare, Chicago, IL, USA) at Epworth Hospital. Following the acquisition, data were pushed to the iCoreLab server where they were automatically moved to reporting or analysis environments based on the receipt of valid clinical details (clinical data) or verified consent (research data). Detailed acquisition protocols are provided in the Supplementary Material Section S1.

### 3.2. Clinical Reporting

Each patient’s CMR exam was clinically reported by CMR experts as per ‘standard of care’, unblinded to patient and clinical information, using all available data obtained from both CMR_FAST_ and CMR_STD_ acquisitions, and adhering to local reporting standards. Clinical measurements were extracted from clinical reports, anonymized, and used as a secondary reference to aid in the evaluation of the standardised research analysis. Details on CMR reporting and image analysis environment are provided in the Supplementary Materials Section S2.

### 3.3. Analytic Strategy

Analyses of 4DCarE imaging data will be conducted in five phases:

Phase 1—Pilot analysis of AscAo and MPA flows comparing 2D-PC and 4D-flow:

- This phase includes the initial 25% of the recruited 4DCarE cohort at the Sydney sites and the results are reported in this paper.
- The main focus is to perform a pilot validation of 4D-flow measurement of AscAo and MPA flows against 2D-PC measurements and compare the measurement accuracy of trained annotators against CMR experts to evaluate the feasibility of the standardised contouring process.
- Lessons learned will help improve the analytical approach in subsequent phases.

Phase 2—Analysis of aortic and pulmonary flows comparing 2D-PC and 4D-flow:

- This phase will test the hypothesis that 4D-flow is non-inferior to 2D-PC for quantification of aortic and pulmonary flows powered by a larger analysis cohort.
- The analysis will employ the first 50% of the entire 4DCarE study cohort.
- As a secondary goal, non-CMR specialist annotators will be trained using standardised contouring protocols developed from Phase 1 feasibility analysis. The performance of the trained annotators will be formally evaluated against CMR experts to demonstrate the feasibility of training annotators to perform at clinically acceptable levels of accuracy and reproducibility.

Phase 3—Analysis of cardiac volume quantification comparing SAX cine and 3D-cine:

- The aim of this phase is to test the hypothesis that 3D-cine is non-inferior to SAX cine for the quantification of cardiac volumes powered by a larger analysis cohort.
- The analysis will employ the first 50% of the entire 4DCarE study cohort.
- As a secondary goal, non-CMR specialist annotators will be trained using standardised contouring protocols. The performance of the trained annotators will be formally evaluated against CMR experts to demonstrate the feasibility of training annotators to perform at clinically acceptable levels of accuracy and reproducibility.

Phase 4—Analysis of the functional characterisation of the whole heart comparing the CMR_FAST_ and CMR_STD_ datasets:

- The aim of this phase is to ascertain the non-inferiority of CMR_FAST_ to CMR_STD_ in the cardiac function diagnostic evaluation, intention-to-treat analysis, and subgroup analyses stratified by diagnostic and severity categories.
- The analysis will include the entire 4DCarE study cohort, divided evenly into test and replication cohorts. The separation of Phase 4 from Phases 2 and 3 and the exclusive use of the replication cohort only in Phase 4 will enable the use of the test cohort for optimising the standardised contouring training process and the replication cohort for repeating the validation and non-inferiority testing.
- A secondary analysis will formally evaluate the benefits and shortcomings of the CMR_FAST_ approach, including ascertaining any clinical or imaging parameters that may limit the clinical use of CMR_FAST_.
- The feasibility and any clinical or imaging limitations of using trained annotators to perform standard contouring will also be formally evaluated.

Phase 5—Novel functional quantification approaches using 3D-cine and 4D-flow MRI:

- Recognising the potential of 4D-flow MRI to derive novel flow parameters to quantify cardiac function [13,14], this phase will aim to investigate the clinical utility of some of these parameters. Potential studies may include but are not limited to (1) wall shear stress analysis in patients with aortic valve pathology or aortopathy [16]; (2) transaortic flow kinetic energy quantification to risk-stratify pathological aortic valve impact on LV dysfunction; and (3) LV vorticity to quantify and risk-stratify early diastolic dysfunction [17].
- Three-dimensional cine provides a structural whole heart measurement that is suitable for automated approaches to evaluating a global function and regional wall motion abnormalities. Volumetric data are also suitable for more direct use in whole-heart modelling approaches [18]. Further studies will explore these aspects both in isolation and in combination with other imaging datasets.

### 3.4. Evaluation of Image Quality

A representative sample of imaging studies was randomly selected from the available total population with a confidence interval of 95% and standard error of 5% for the evaluation of quality by two CMR experts, both with Society of Cardiovascular Magnetic Resonance (SCMR) Level 3 or equivalent qualifications. Standardised quality evaluation criteria were developed [19] to rate the imaging studies as good, moderate, or poor quality, with criteria including completeness of series, coverage of the region of interest, presence of artefacts, signal loss, eddy current, and velocity aliasing. For each imaging study, 2D-PC, 4D-flow, 3D-cine, and SAX series were evaluated together to form a quality assessment. Imaging studies rated as poor were deemed not suitable for inclusion in the flow and volume analysis.

### 3.5. Image Analysis

Image analysis was performed by two CMR experts, both with SCMR Level 3 or equivalent qualifications, and non-CMR trained annotators who have undergone an in-house standardised training process. The measurements performed by the two CMR experts on the same cases were compared, and measurement techniques were refined to determine a ‘gold standard’ contouring approach. For the training of the annotators, an application-specific radiology anatomy atlas was developed to teach annotators to recognise key anatomical landmarks and standard radiological views. A codified contouring protocol was developed for each SAX cine, 2D-PC, 3D-cine, and 4D-flow contouring. Annotators had basic health sciences or medical backgrounds but no prior dedicated CMR training. Each annotator underwent an initial training set of 20 cases preselected at random with answers available on completion and no limitation on the number of attempts. Following that, a further 20 preselected random cases were contoured using a single attempt, and the annotator was blinded from the measurement results. The annotator’s measurements were compared with those obtained by the CMR experts and analysed using the intraclass correlation coefficient (ICC) and Bland–Altman plot. Satisfactory performance was determined as ICC > 0.90, mean difference (MD) less than 5 mL/beat, and reproducibility coefficient (RPC) less than 10 mL/beat for flow and volume measurements [20]. The training process was repeated until satisfactory performance was achieved. The pilot analysis was performed by one of the CMR experts in AscAo and MPA and was internally blinded, i.e., the measurements of 2D-PC and 4D-flow were performed independently of each other. Two-dimensional PC measurements were performed first for the entire pilot cohort, followed by 4D-flow measurements. Specific analysis details for 2D-PC and 4D-flow are provided in the Supplementary Materials Section S3.

### 3.6. Power Calculations

Based on our own previous data, the mean pulmonary flow from the 2D-PC measurement was expected to be approximately 95 mL/beat, and the aortic flow was expected to be approximately 84 mL/beat, with standard deviations (SD) of around 30 mL/beat for both measures. The values were expected to be similar to 4D-flow measurements. The SD for 4D-flow-derived flows across the population was expected to be reduced (approximately half of that of 2D-PC in a previous cohort); however, this would be greatly affected by the makeup of the cohorts and, hence, the distribution of actual pulmonary or aortic flows [18]. The ventricular volume measurements using 3D-cine and SAX cine were likely to be similar. The SD of volume measurements would be similar to that for flow measurements.

All power calculations were performed according to the methodology of Lui et al. [21] where a non-inferiority margin of 10% was arbitrarily set and an SD of 30% was used (based on the approximate measured SD across a population of 150 general subjects from conventional flow in a prior cohort). Flow data were used for power calculations since this was expected to be the noisiest measured primary outcome variable. For a statistical power of 0.90 and an alpha or *p*-value (type one error) of 0.025, a sample size of 378 would be required to determine non-inferiority.

### 3.7. Statistical Analysis

Intra- and inter-observer reproducibility was analysed using ICC, Pearson’s correlation coefficient ($r^{2}$), and Bland–Altman plot. No significant systematic bias was assumed if MD between two measurements was less than 5 mL/beat, the 95% confidence interval (CI) of the intercept included the value 0, and RPC was less than 10 mL/beat. An agreement between 2D and 3D techniques was analysed using the same statistical methods as in reproducibility analysis. For ICC, an agreement was considered poor, moderate, good, or excellent for ICC < 0.50, 0.50–0.75, 0.75–0.90, and >0.90, respectively. Where applicable, statistical significance was set at *p* < 0.05.

## 4. Results

Here, we present an overall appraisal of the pilot cohort validation of 4D-flow against 2D-PC flow measurements in the AscAo and MPA, together with an evaluation of our initial experience with standardised image analysis and annotator training for 2D-PC and 4D-flow datasets.

### 4.1. Clinical Characteristics and Data Quality—Pilot Cohort

#### 4.1.1. Clinical Characteristics

Table 1 summarises the clinical characteristics of the pilot cohort. Overall, the cohort represented a diverse range of cardiac volumes, functions, and indications representing both acquired and congenital cardiac pathology. A total of 20% of the cohort had some degree of aortic regurgitation, 8.2% had aortopathy, 5.6% had established valvular disease, and 3.6% were imaged to evaluate shunt.

#### 4.1.2. Evaluation of Imaging Quality

**Figure 2:**
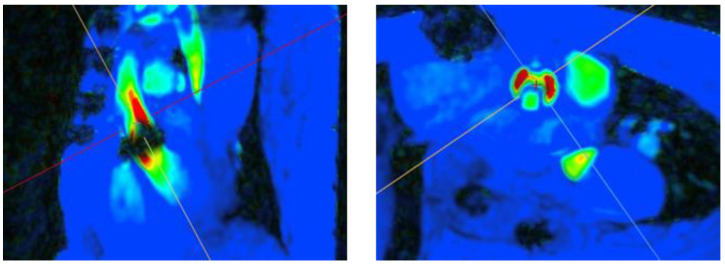
The presence of implanted aortic valve (**left**) resulted in a flow pattern artefact in the ascending aorta (**right**). Color heatmap corresponds with flow velocity (red indicates high flow regions, green indicates low flow regions, blue indicates background static tissue). The cross (+) indicates the centre of regional of interest and the perpendicular lines are used to orientate the regional of interest.

**Figure 3:**
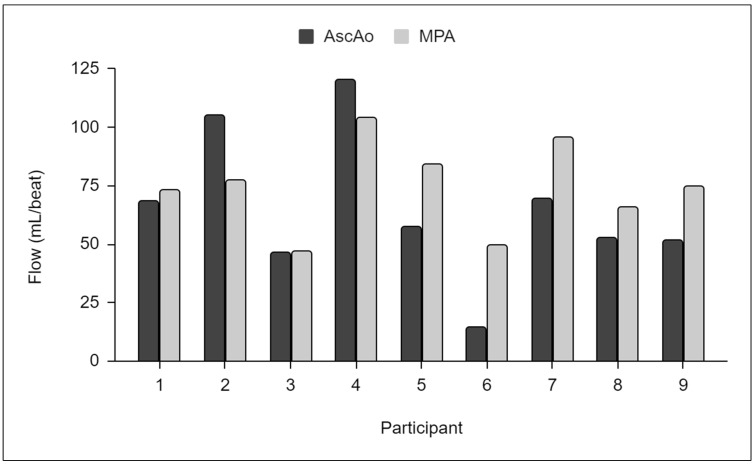
Flow measurements from nine poor-quality 4D-flow series showing discordant flows between the ascending aorta (AscAo) and main pulmonary artery (MPA).

A sample of 250 imaging studies from an available population of 1150 studies were selected for quality evaluation. A total of 92% of the evaluated studies were rated as good or moderate, and 8% were rated as poor. In the poor-quality studies, 4D-flow was the main contributor to poor-quality ratings, predominantly due to motion artefact/mistriggering (40%) and velocity aliasing (45%). Significant metallic artefacts was present in 25% of the poor-quality studies, secondary to the presence of sternotomy wires or implanted cardiac devices (Figure 2). Thirty-two percent of the poor-quality studies were also missing the correct 4D-flow series. Flow measurements were impacted by imaging quality. Discordant flows were observed between AscAo and MPA in nine poor-quality studies where measurements could be obtained (Figure 3).

### 4.2. Intra-/Inter-Observer Reproducibility

Two CMR experts undertook image analysis on 2D-PC and 4D-flow images independently on 19 cases measuring flow in AscAo and MPA, both blinded to each other’s measurements. Each expert also repeated their measurements on the same cases one month after the initial measurements. The second measurement was also internally blinded from the first measurement.

Table 2 summarises the intra-observer reproducibility for both experts. Both experts demonstrated a high degree of reproducibility for both techniques measuring in the AscAo and MPA with ICC > 0.90 for all measurements except for 4D-flow measurements of backward flow in AscAo (Expert 1: ICC 0.72 [95% CI 0.35–0.90], MD = −0.12 ± 1.42 mL/beat, RPC = 2.78 mL/beat; Expert 2: ICC 0.60 [95% CI 0.15–0.84], MD = 0.06 ± 2.08 mL/beat, RPC = 4.08 mL/beat); however, the magnitudes of backward flow were negligible in the absence of regurgitation and would be more likely influenced by signal noise and not likely to be clinically significant.

### 4.3. Inter-Observer Reproducibility

Table 3 summarises the inter-observer reproducibility between the two CMR experts. An agreement between the two experts was very high (ICC > 0.9) across measurements, except for 4D-flow measurements of backward flow (AscAo: ICC 0.84 [95% CI 0.59–0.94], MD = −0.21 ± 0.82 mL/beat; MPA: ICC 0.88 [95% CI 0.68–0.96], MD = 0.34 ± 1.39 mL/beat). This, however, was a small absolute difference (AscAo: RPC = 1.62 mL/beat; RPC = 2.73 mL/beat) and is unlikely to be clinically significant.

### 4.4. Pilot Cohort Analysis

**Figure 4:**
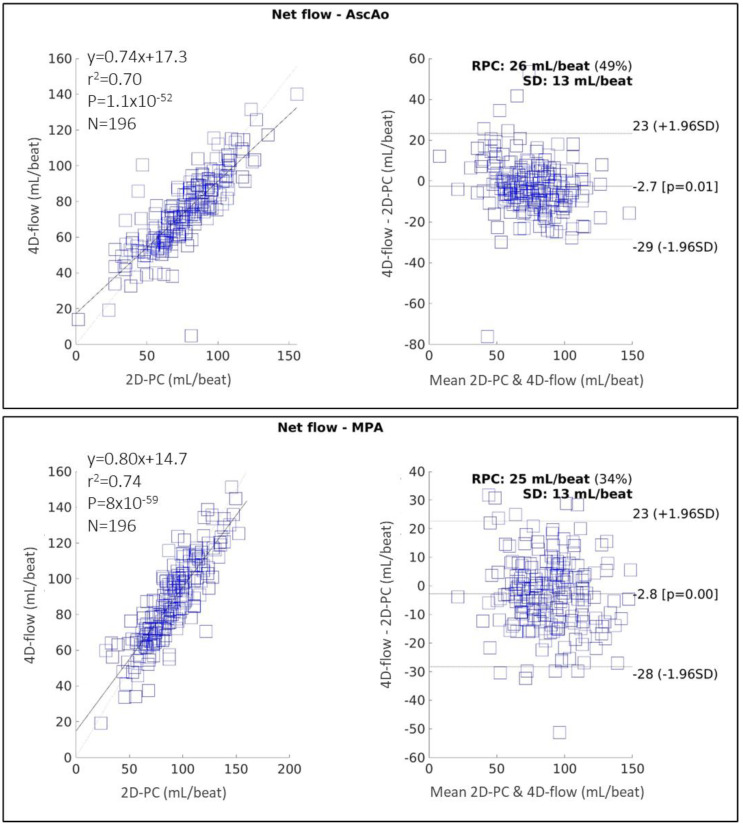
Correlation and Bland–Altman plots comparing measurements in the ascending aorta (AscAo) (top) and main pulmonary artery (MPA) between 2D-PC and 4D-flow performed by a CMR expert on a pilot cohort of 196 cases. RPC: reproducibility coefficient; SD: standard deviation.

Figure 4 shows the correlation and Bland–Altman plots of 2D-PC and 4D-flow measurements performed by an expert annotator in the AscAo and MPA for the pilot cohort of 196 cases. For both vessels, 4D-flow underestimated the flow compared with 2D-PC by approximately 3 mL/beat (or 1.96% for AscAo and 2.89% for MPA). In the AscAo, the inter-technique agreement was moderate–good (ICC 0.83 [95% CI 0.77–0.87], $r^{2}$ = 0.70, MD = −2.7 ± 13 mL/beat, *p* = 0.01, RPC = 26 mL/beat). In the MPA, the inter-technique agreement was also moderate–good (ICC 0.85 [95% CI 0.80–0.89], $r^{2}$ = 0.74, MD = −2.8 ± 13 mL/beat, *p* = 0.002, RPC = 25 mL/beat). Outliers were identified as cases lying outside of the limit of agreement (LoA) and were individually reviewed. Out of 196 cases, 30 cases were identified as outliers, representing approximately 15% of the cohort. The main contribution to outliers was found to be secondary to anterior–posterior (A-P) ghosting in either the 2D-PC or 4D-flow images, resulting in poor image quality. Other reasons for outliers included inaccurate placement of 2D-PC acquisition planes and significantly different heart rates between 2D-PC and 4D-flow acquisitions in the same participant. Whilst the outliers were included in the results on an intention-to-treat basis, they represented the potential system limitations of either modality and contributed to the understanding of the likely proportion of cases that could be suboptimally measured by either modality in the overall 4DCarE cohort and real-world clinical practice. Despite these issues, the bias of approximately 3 mL/beat was well within our predefined non-inferiority margin of 10%.

### 4.5. Standardisation of Image Analysis Process—Initial Experience

#### Application of Standard Contouring Protocols

As part of the secondary study goal, we sought to evaluate whether trained annotators could perform image contouring at a clinically acceptable standard by undertaking a standardised training process with codified contouring protocols. The development of the protocols was an iterative process, and the results shown here are the outcomes from the latest iteration. Annotators were always blinded to case identification and were presented with fresh data at each iteration to avoid bias during training. A sample of 100 randomly selected cases were used for training of the annotators.

2D-PC

**Figure 5:**
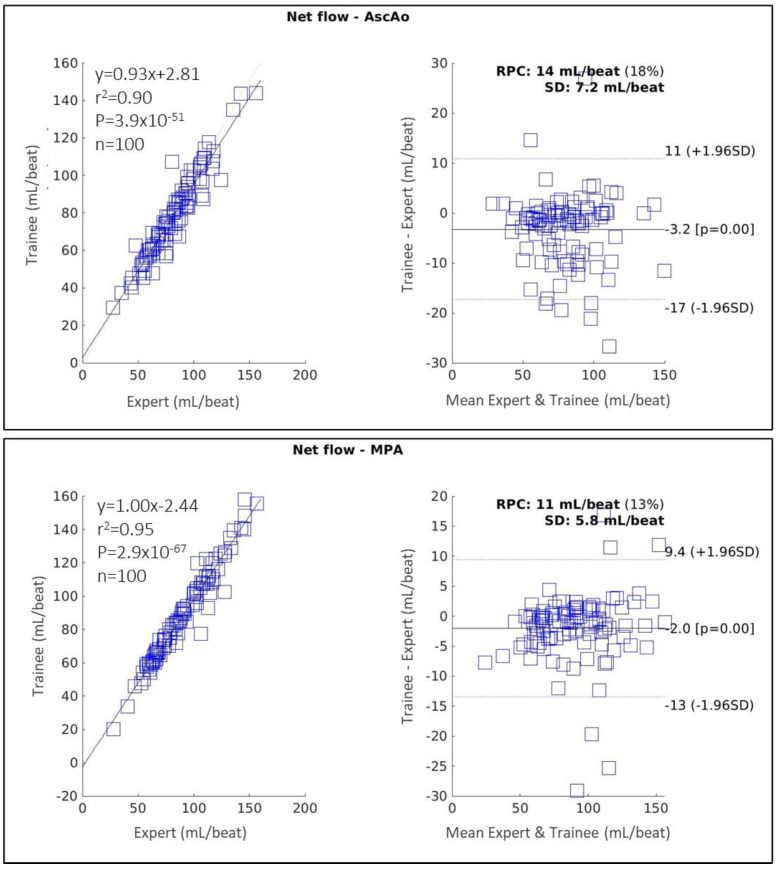
Correlation and Bland–Altman plots showing 2D-PC measurements in the ascending aorta (AscAo) (top) and main pulmonary artery (MPA), comparing a CMR expert with a trained annotator. RPC: reproducibility coefficient; SD: standard deviation.

Figure 5 shows the correlation and Bland–Altman plots for net flow measured in the AscAo and MPA using 2D-PC to compare a trained annotator with a CMR expert. For measurements in AscAo, the trained annotator’s performance was rated good (ICC 0.93 [95% CI 0.90–0.95], $r^{2}$ = 0.90, MD = −3.2 ± 7.2 mL/beat, *p* < 0.05, RPC = 14 mL/beat). In the MPA, the trained annotator’s performance was excellent, (ICC 0.97 [95% CI 0.96–0.98], $r^{2}$ = 0.95, MD = −2.0 ± 5.8 mL/beat, *p* < 0.05, RPC = 11 mL/beat).

4D-flow

**Figure 6:**
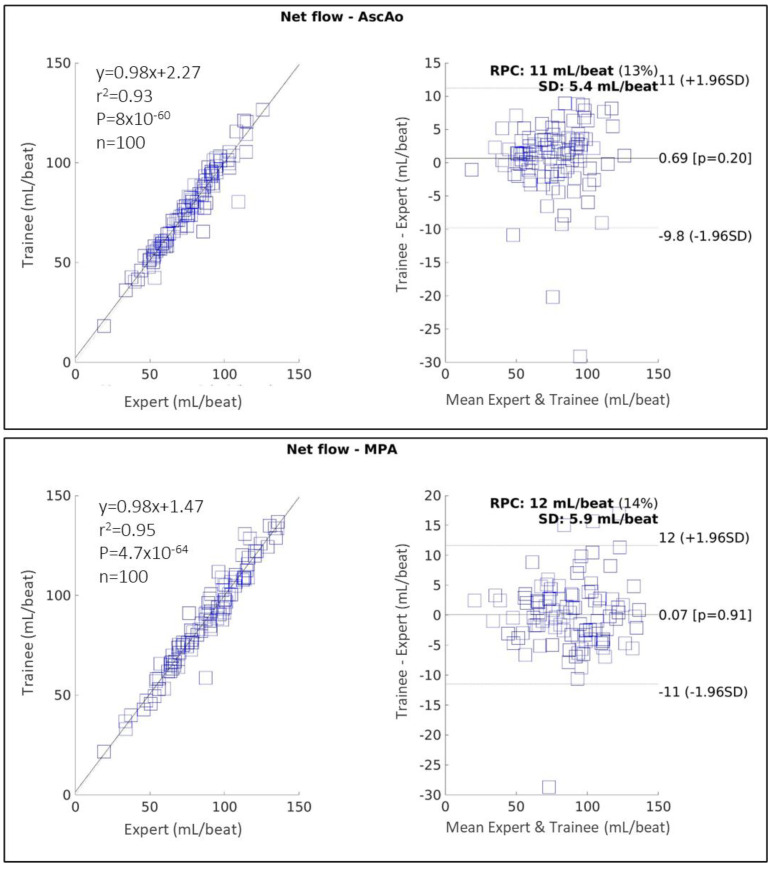
Correlation and Bland–Altman plots showing 4D-flow measurements in the ascending aorta (AscAo) (top) and main pulmonary artery (MPA), comparing a CMR expert with a trained annotator. RPC: reproducibility coefficient; SD: standard deviation.

Figure 6 shows the correlation and Bland–Altman plots for the net flow measured in the AscAo and MPA using 4D-flow, comparing a trained annotator with a CMR expert. In AscAo, the performance of the annotator was excellent (0.97 [95% CI 0.95–0.98], $r^{2}$ = 0.93, MD = 0.69 ± 5.37 mL/beat, *p* = 0.20, RPC = 10.5 mL/beat). Performance in the MPA was also excellent (ICC 0.97 [95% CI 0.96–0.98], $r^{2}$ = 0.95, MD = 0.07 ± 5.90 mL/beat, *p* = 0.91, RPC = 11.6 mL/beat).

**Figure 7:**
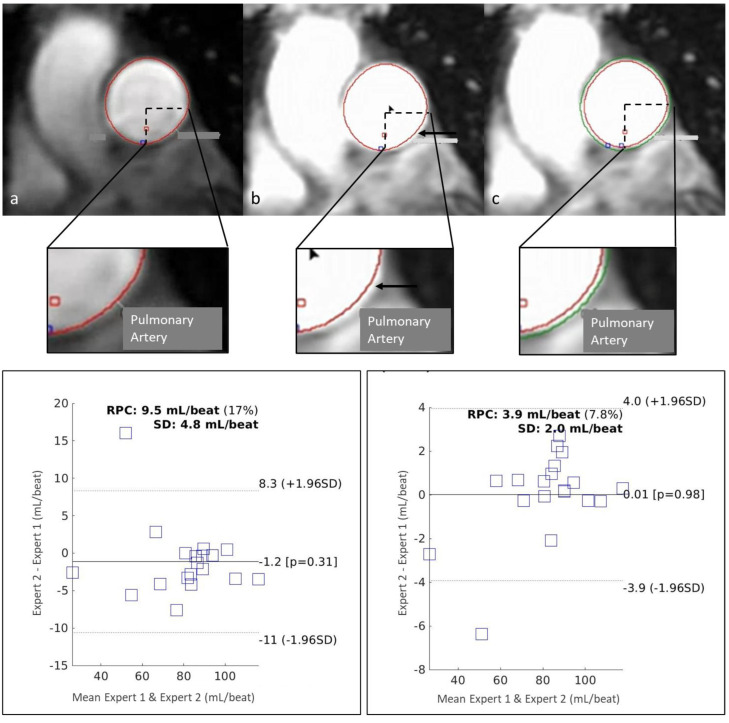
The top row (and magnified views): vessel boundary definition in 2D-PC contouring. (**a**) Initial contouring approach (red contour); (**b**) adjustment of windowing revealing pixels adjacent to the vessel wall excluded from the contour (black arrow); (**c**) a revised approach with expanded contour [24] (green contour) capturing all flow signal-containing pixels. The bottom row: Bland–Altman plots showing inter-observer reproducibility between two CMR experts using an unstandardised contouring approach (left) and a standardised expanded contouring approach (right), showing improved reproducibility following standardisation. RPC: reproducibility coefficient; SD: standard deviation.

A key early lesson learned from the 2D-PC contouring process was the influence of the vessel boundary definition on flow measurement. Due to the partial volume effect on vessel wall boundaries [22,23], an initial approach to vessel contouring resulted in numerically lower estimates of flow due to the exclusion of pixels adjacent to the vessel walls, which partially contained blood flow signals. The adjustment of windowing revealed these adjacent-wall pixels, and a revised, radially expanded contour resulted in increased measured flow, by approximately 2%, and improved inter-observer reproducibility with a mean difference between the two CMR experts decreasing from 1.2 mL/beat (or 1.2%) to 0.01 mL/beat (or 0.6%) and RPC decreasing from 9.5 mL/beat to 3.9 mL/beat (Figure 7) [24].

**Figure 8:**
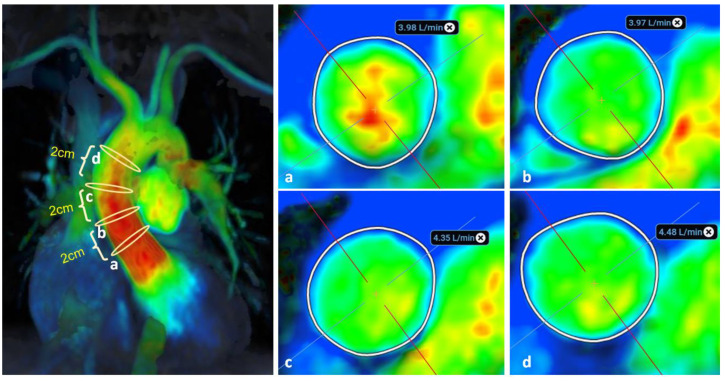
Impact of analysis location on the 4D-flow measurement of flow in AscAo: (**a**–**d**) four analysis locations (positions a–d from proximal to distal relative to the aortic valve, 2 cm apart as outlined on the velocity magnitude colour map. Net flow measured in L/min. Color heatmap corresponds with flow velocity (red indicates high flow regions, green indicates low flow regions, blue indicates background static tissue). The cross (+) indicates the centre of regional of interest and the perpendicular lines are used to orientate the regional of interest.

**Figure 9:**
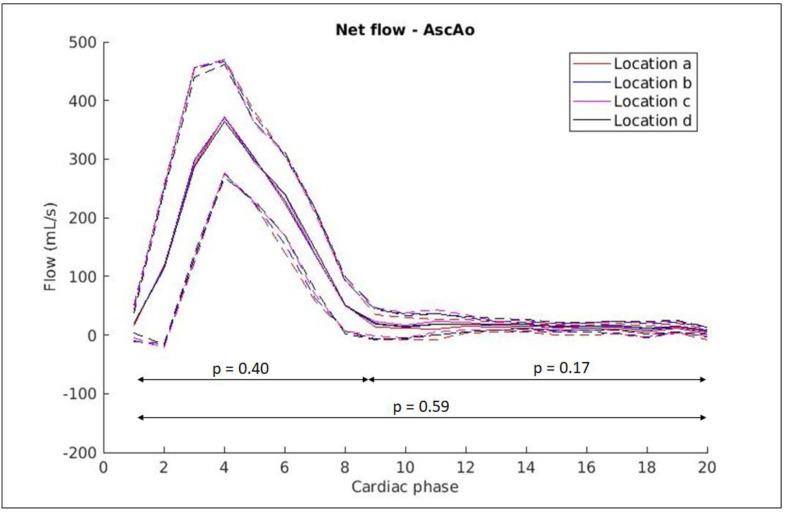
CMR expert-measured flows at locations a–d along the ascending aorta, with flow curves showing median (solid lines) and one standard deviation (dotted lines) values for 20 participants. Repeated measures ANOVA was performed on the full cardiac cycle (*p* = 0.59), systolic phase (*p* = 0.40) and diastolic phase (*p* = 0.17). The measurements at the four locations are very similar as illustrated by the lines overlapping one another.

It has been theorised that the predisposition for the formation of strong, asymmetrical vortices or recirculation zones in the ascending aorta potentially has an impact on the precision and reproducibility of flow measurements [25]. The presence of strong zones of turbulent flow may exacerbate flow measurement differences from ROI placement on 4D-flow images between individual annotators. For example, an annotator’s selected analysis location may be relatively proximal or distal to or centred within the region of maximal turbulence (Figure 8) [24]. In our initial analysis, measurements at four locations along the ascending aorta were obtained by a CMR expert in 20 healthy participants. There were no statistically significant differences between measurements obtained at the locations (Figure 9). It is possible that flow in the healthy aorta of the 20 participants was predominantly lamina, and the differences between measurement locations could become more apparent in pathological cases where flow tends to be turbulent.

## 5. Discussion

4DCarE is a prospective multi-centre non-inferiority study that addresses the need for more rapid CMR acquisitions and standardised image analysis approaches in order to reduce the cost, time, and expertise necessary to perform a CMR exam without the loss of accuracy. We presented the study protocol of 4DCarE and supported it with a pilot cohort analysis of 4D-flow and 2D-PC measurements. Additionally, we described our initial experience with a standardized image analysis approach with an embedded non-CMR expert annotator training process to reduce the resource burden of CMR analysis. The study protocol leveraged the existing capabilities at the participating imaging centres and was time-neutral and noninterventional. The anticipated cohort of 1200 participants is the largest reported in similar studies.

The pilot analysis of the initial 25% of the recruited cohort demonstrated moderate–good agreement of flow measurements between 2D-PC and 4D-flow in line with consensus recommendation [8]. Whilst not powered to test non-inferiority with this pilot cohort, the results lend confidence to our analysis methodology and the likely positive outcomes in our future phases of study.

Our initial experience with standardisation of the image analysis process and training of non-CMR expert annotators demonstrated the potential for the routine use of non-CMR accredited trained annotators to perform objective and accurate image analysis at a clinically acceptable level, potentially reducing cost and workload burden on expert clinicians [15].

A key error source in 2D-PC contouring originated from vessel boundary definition. The optimisation of windowing and standardisation of the contouring approach to incorporate adjacent-wall pixels improved inter-observer consistency in flow measurements.

Four-dimensional flow measurements were not found to be impacted by analysis plane positioning along the ascending aorta in 20 healthy subjects. However, this could potentially be due to the predominantly uniform laminar flow in healthy aortas. A bigger impact may be observed in pathological cases where flow is more likely to be turbulent along the aorta.

An additional consideration is the fraction of clinical cases that are likely only suitable for a CMR expert to analyse. This may be relevant to clinical translation and is likely to be different for 2D and 3D images. Current case numbers are underpowered to ascertain the parameters defining these cases (especially when different diagnostic categories are considered), and we will aim to report our further findings in the subsequent phases of the study.

Future planned and exploratory analyses of the full dataset will likely yield additional valuable lessons. Exploration of the wealth of information available from 3D imaging techniques will create opportunities to define and evaluate cardiac function with a whole suite of novel parameters [16,17]. Leveraging the size of our target study cohort, we aim to systematically investigate the clinical utilities of some of the 4D-flow-derived parameters in a number of sub-studies, which may include but not be limited to wall shear stress (WSS), transaortic flow kinetic energy (KE), and LV vorticity.

WSS is the tangential viscous shear force per unit area exerted on the blood vessel wall by the adjacent fluid layer [13]. Altered WSS on the aorta is known to be associated with aortopathy and aortic valve pathologies and may be useful in predicting aortic dilatation [26]. Quantification of WSS in the presence and absence of aortic valvular disease may be helpful in defining WSS as a clinical parameter that directly links valvular disease severity to the risk of developing aortic dilatation [16].

Transaortic flow kinetic energy may be another functional parameter that links aortic valve disease to the risk of LV remodelling and dysfunction. Aortic stenosis or regurgitation is known to cause LV remodelling and eventual failure in the long term. Early subtle KE changes may be present before any obvious remodelling or dysfunction has occurred [27]. Quantifying KE of flow across the aortic valve may be a way to risk-stratify LV dysfunction in the presence of valvular disease.

LV diastolic vortex formation is known to be an important natural phenomenon impacting cardiac function and efficiency. Alterations in the vortex formation and vorticity were shown to be associated with diastolic dysfunction even in the absence of clinically evident LV remodelling. However, there has not been a systematic evaluation of LV vorticity to risk-stratify LV dysfunction [17]. There remains the potential to evaluate this novel parameter as a marker for early diastolic dysfunction before any clinically evident remodelling or failure has occurred.

The clinical implications of 4DCarE is potentially wide-ranging. If the study demonstrates non-inferiority of the rapid CMR protocol compared with conventional CMR, it can potentially support an increased CMR use in routine clinical workflow. Should the study prove that trained annotators can perform image analysis to clinically acceptable standards, it will potentially reduce the labor and cost requirements of image analysis and further support the wider clinical adoption of CMR. Investigating the 3D imaging-derived parameters can potentially identify new clinically relevant cardiac function measures that may help improve the diagnosis and assessment of cardiac pathologies.

## 6. Conclusions

4DCarE is a prospective multi-centre study aiming to investigate the non-inferiority of 3D volumetric CMR acquisition techniques (3D-cine and 4D-flow) compared with conventional 2D techniques (SAX cine and 2D-PC). We presented the study protocol, supported by the pilot analysis of an initial 196 cases, which found a good agreement of flow measurements between 2D-PC and 4D-flow. Initial experience with training non-CMR expert annotators to perform standardized image analysis showed promise that the annotators can potentially perform at clinically acceptable standards. Subsequent phases of the study will report on the analysis performed on the full study cohort, as well as sub-studies investigating the clinical applicability of 4D-flow-derived novel flow parameters in quantifying cardiac function. 4DCarE will potentially have a wide range of clinical implications that may support the broader clinical adoption of a rapid CMR protocol.

## Figures and Tables

**Table 1:** Clinical characteristics of the pilot cohort. EF: ejection fraction; EDV: end-diastolic volume; LV: left ventricle; RV: right ventricle; SV: stroke volume; AscAo: ascending aorta; MPA: main pulmonary artery; ARVC: arrhythmogenic right ventricular cardiomyopathy.

*n* = 196			
Basic Clinical Parameters	Mean ± Std (Range)	Indication	% Total
Age (years)	54 ± 18 (16–89)	Cardiomyopathy	26.0%
Female (%)	46.1%	Viability	11.2%
LVEF (%)	60 ± 14% (15–86%)	ARVC	10.7%
LVEDV (mL)	153.03 ± 43.40 (67–297)	Perimyocarditis	9.2%
LVSV (mL)	88.50 ± 23.52 (37–179)	Aortopathy	8.2%
RVEF (%)	59 ± 9% (31–86%)	Hypertrophic cardiomyopathy	7.1%
RVEDV (mL)	148.34 ± 43.40 (63–284)	Valvular disease	5.6%
RVSV (mL)	85.26 ± 22.15 (22–148)	Shunt	3.6%
AscAo net flow (mL/beat)	78.56 ± 22.91 (22–142)	Iron overload	3.6%
MPA net flow (mL/beat)	85.27 ± 24.12 (26–150)	Congenital	3.6%
		Infiltration	3.6%
Aortic regurgitation grade	% total	Arrythmia	2.6%
None	80.0%	Cardiac tumour	1.5%
Trivial	9.1%	Heart failure	1.0%
Mild	7.3%	Pulmonary hypertension	1.0%
Moderate	3.6%	Right heart function	1.0%
		Pulmonary Regurgitation	0.5%

**Table 2:** Intra-observer reproducibility of two CMR experts (Measurements 1 and 2 denote two attempts performed one month apart). AscAo: ascending aorta; ICC: intraclass correlation coefficient; MD: mean difference; MPA: main pulmonary artery; RPC: reproducibility coefficient.

**Expert 1**					
***n* = 19**	**2D-PC**				
	**Measurement 1**	**Measurement 2**	**ICC** **(95% CI)**	**MD ± Std**	**RPC**
**AscAo**	(mean ± std), mL/beat			mL/beat	mL/beat
Forward flow	85.45 ± 19.93	85.63 ± 19.40	1.00 (0.99–1.00)	0.18 ± 1.49	2.92
Backward flow	4.34 ± 4.64	3.85 ± 3.84	0.95 (0.87–0.98)	−0.49 ± 1.23	2.41
Net flow	81.11 ± 21.19	81.78 ± 20.12	0.99 (0.98–1.00)	0.66 ± 2.55	5.00
**MPA**					
Forward flow	98.42 ± 19.94	97.33 ± 19.53	0.99 (0.98–1.00)	−1.09 ± 1.85	3.63
Backward flow	1.99 ± 2.58	1.90 ± 2.54	1.00 (0.99–1.00)	−0.09 ± 0.17	0.34
Net flow	96.43 ± 19.56	95.43 ± 19.12	0.99 (0.98–1.00)	−0.99 ± 1.87	3.67
***n* = 19**	**4D-flow**				
	**Measurement 1**	**Measurement 2**	**ICC** **(95% CI)**	**MD ± Std**	**RPC**
**AscAo**	(mean ± std), mL/beat			mL/beat	mL/beat
Forward flow	82.08 ± 17.49	83.53 ± 17.40	0.98 (0.94–0.99)	1.45 ± 3.50	6.86
Backward flow	1.29 ± 1.47	1.17 ± 2.17	0.72 (0.35–0.9)	−0.12 ± 1.42	2.78
Net flow	80.80 ± 17.33	82.36 ± 17.13	0.96 (0.90–0.99)	1.56 ± 4.46	8.75
**MPA**					
Forward flow	95.21 ± 20.51	93.51 ± 18.26	0.97 (0.91–0.99)	−1.70 ± 4.83	9.46
Backward flow	1.54 ± 2.21	1.84 ± 3.35	0.88 (0.69–0.96)	0.30 ± 1.39	2.73
Net flow	93.67 ± 19.93	91.67 ± 17.60	0.95 (0.86–0.98)	−2.00 ± 5.82	11.41
**Expert 2**					
***n* = 19**	**2D-PC**				
	**Measurement 1**	**Measurement 2**	**ICC** **(95% CI)**	**MD ± Std**	**RPC**
**AscAo**	(mean ± std), mL/beat			mL/beat	mL/beat
Forward flow	83.24 ± 19.66	82.97 ± 19.69	1.00 (0.99–1.00)	−0.26 ± 1.62	3.18
Backward flow	3.27 ± 3.11	3.45 ± 3.56	0.96 (0.89–0.99)	0.18 ± 0.94	1.85
Net flow	79.96 ± 20.15	79.52 ± 20.35	1.00 (0.99–1.00)	−0.44 ± 1.85	3.63
**MPA**					
Forward flow	93.35 ± 19.99	92.03 ± 19.73	0.99 (0.97–1.00)	−1.32 ± 2.31	4.53
Backward flow	1.74 ± 2.40	1.71 ± 2.40	1.00 (1.00–1.00)	−0.03 ± 0.08	0.16
Net flow	91.62 ± 19.50	90.33 ± 19.19	0.99 (0.97–1.00)	−1.29 ± 2.27	4.46
***n* = 19**	**4D-flow**				
	**Measurement 1**	**Measurement 2**	**ICC** **(95% CI)**	**MD ± Std**	**RPC**
**AscAo**	(mean ± std), mL/beat			mL/beat	mL/beat
Forward flow	83.70 ± 17.25	82.54 ± 17.73	0.95 (0.87–0.98)	−1.16 ± 5.40	10.59
Backward flow	1.02 ± 2.85	1.08 ± 1.46	0.60 (0.15–0.84)	0.06 ± 2.08	4.08
Net flow	82.68 ± 17.20	81.46 ± 17.55	0.93 (0.80–0.97)	−1.22 ± 6.71	13.16
**MPA**					
Forward flow	91.17 ± 18.41	94.09 ± 18.76	0.96 (0.89–0.99)	2.92 ± 4.37	8.56
Backward flow	1.79 ± 2.85	1.88 ± 3.31	0.97 (0.91–0.99)	0.09 ± 0.78	1.53
Net flow	89.38 ± 17.87	92.22 ± 18.17	0.96 (0.88–0.98)	2.83 ± 4.67	9.15

**Table 3:** Inter-observer reproducibility between two CMR experts. AscAo: ascending aorta; ICC: intraclass correlation coefficient; MD: mean difference; MPA: main pulmonary artery; RPC: reproducibility coefficient.

***n* = 19**	**2D-PC**				
	**Expert 1**	**Expert 2**	**ICC** **(95% CI)**	**MD ± Std**	**RPC**
**AscAo**	(mean ± std), mL/beat			mL/beat	mL/beat
Forward flow	85.63 ± 19.40	85.83 ± 20.23	1.00 (0.99–1.00)	0.20 ± 1.99	3.91
Backward flow	3.85 ± 3.84	4.04 ± 3.70	0.99 (0.97–1.00)	0.19 ± 0.49	0.95
Net flow	81.78 ± 20.12	81.79 ± 21.13	1.00 (0.99–1.00)	0.01 ± 2.01	3.95
**MPA**					
Forward flow	97.33 ± 19.53	99.06 ± 20.37	0.99 (0.97–1.00)	1.73 ± 2.33	4.56
Backward flow	1.90 ± 2.54	1.83 ± 2.38	0.99 (0.98–1.00)	−0.08 ± 0.27	0.52
Net flow	95.43 ± 19.12	97.24 ± 19.86	0.99 (0.97–1.00)	1.80 ± 2.46	4.82
***n* = 19**	**4D-flow**				
	**Expert 1**	**Expert 2**	**ICC** **(95% CI)**	**MD ± std**	**RPC**
**AscAo**	(mean ± std), mL/beat			mL/beat	mL/beat
Forward flow	82.08 ± 17.49	82.54 ± 17.73	0.99 (0.96–0.99)	0.46 ± 3.09	6.05
Backward flow	1.29 ± 1.47	1.08 ± 1.46	0.84 (0.59–0.94)	−0.21 ± 0.82	1.62
Net flow	80.80 ± 17.33	81.46 ± 17.55	0.98 (0.94–0.99)	0.67 ± 3.74	7.32
**MPA**					
Forward flow	95.21 ± 20.51	94.09 ± 18.76	0.99 (0.97–1)	−1.11 ± 2.87	5.63
Backward flow	1.54 ± 2.21	1.88 ± 3.31	0.88 (0.68–0.96)	0.34 ± 1.39	2.73
Net flow	93.67 ± 19.93	92.22 ± 18.17	0.98 (0.94–0.99)	−1.45 ± 3.78	7.40

## Data Availability

All data generated or analysed during this study are included in this published article.

## Supplementary Materials

The following supporting information can be downloaded at: https://www.mdpi.com/article/10.3390/diagnostics14222590/s1, Section S1: Detailed CMR acquisition protocols; Section S2: CMR Reporting and Image Analysis Environment; Section S3: Specific image analysis details; Figure S1: Data flow process for clinical reporting and research analysis. PWD: pulsed wave doppler; SAX: short-axis; Figure S2: Typical 2D-PC contouring in CVI42. (a) contouring of regional of interest (ROI); (b) numerical outputs calculated from ROIs; (c) flow curve calculated from ROIs. AscA: ascending aorta; MPA: main pulmonary artery; Figure S3: Typical 4D-flow contouring in Arterys. (a) whole-heart visualisation for orientation; (b) numeric outputs; (c–h) 3-plane contouring views for the ascending aorta (AscAo) (c–e) and pulmonary artery (MPA) (f–h) with axial (e,f), sagittal (d,g) and coronal (e,h) views; Table S1: Detailed Summary of Acquisition Protocol.

## Abbreviations

2D	two-dimensional
3D	three-dimensional
4D	four-dimensional
AscAo	ascending aorta
AV	aortic valve
CMR	cardiac magnetic resonance
CT	computer tomography
DGE	delayed gadolinium enhancement
EDV	end-diastolic volume
EF	ejection fraction
ESV	end-systolic volume
ICC	intraclass correlation coefficient
KE	kinetic energy
LV	left ventricle
MD	mean difference
MPA	main pulmonary artery
MRA	magnetic resonance angiography
PC	phase contrast
ROI	region of interest
RPC	reproducibility coefficient
SAX	short-axis
SV	stroke volume
TTE	transthoracic echocardiogram
WSS	wall shear stress
