# Supplementary material for: Four-Dimensional Flow MRI for Cardiovascular Evaluation (4DCarE): A Prospective Non-Inferiority Study of a Rapid Cardiac MRI Exam: Study Protocol and Pilot Analysis
Source: Diagnostics (Basel). 2024 Nov 18;14(22):2590. doi: 10.3390/diagnostics14222590 (PMC11593203; doi:10.3390/diagnostics14222590)
Supplement: Supplementary file 1 [file diagnostics-14-02590-s001.zip › diagnostics-3278429-supplementary.pdf]

Supplementary Materials

S1. Detailed CMR acquisition protocols

An 18-channel phased array anterior coil in combination with a linear 12-channel posterior coil was employed at all sites. Both 3D-cine and 4D-flow were already in clinical use at all three sites and were acquired regardless of inclusion in the study. The study protocol was therefore time-neutral compared to the prior standard exam at the participating sites and averaged 50-55min.

Summary of the acquisition parameters is included in Supplemental Material Table S1. All datasets were acquired in a single imaging session with the CMR_{FAST} dataset acquired during the first 15-20min and the CMR_{STD} dataset acquired during the remaining scan time. Any clinically necessary additional sequences were added as per the supervising radiologist at each site. Clinical staff had all the normal access to patient demographic and clinical history at the time of image acquisition, while standardised research analysis was performed blinded to patient and clinical information.

Three planning sequences (3-plane scout, LVLA cine, axial FIESTA stack) were acquired to permit accurate positioning of the 4D-flow, 3D-cine, and Magnetic Resonance Angiography (MRA) volumes. Contrast was then administered, with 3D-cine performed at first pass post-contrast, followed by the MRA and 4D-flow acquisitions. Immediately following 4D-flow acquisition, look-locker was performed to determine the adequate inversion time (TI) to acquire delayed gadolinium enhancement (DGE) images. Additional DGE acquisitions were performed as deemed necessary by the radiographer. 2D-PC, SAX, long-axis cine views and a T2-weighted SAX were sequentially acquired, followed by any other clinically required sequences at the end of the study. The acquisitions of MRA, DGE and T2 images were clinically indicated but do not contribute to the objectives of the study. The acquisition parameters for the key sequences are reported briefly here for completeness.

3D-cine was acquired as a prospectively ECG-gated, single breath-hold sagittal acquisition covering the entire heart and great vessels following a gadolinium administration of 25mL (0.1mmol/kg) injected at 2.5mL/s with a 20sec delay, adjusted to 30sec in the presence of known or suspected moderate-severe heart failure. Parameters: field of view (FOV) 250 × 250mm, voxel size $2mm^3$ isotropic, TE 1.2ms, TR 2.5ms, flip angle 20°, and 20 phases per cardiac cycle. Average breath-hold was 15-17sec.

The 4D-flow acquisition used a 4-point referenced phase-encoding strategy at a subject specific encoding velocity (VENC) of 150 to 170cm/s, determined from 2D-PC measurements made at the ascending aorta (AscAo) (Callaghan et al. 2016). A prospective ECG-gated, free-breathing sagittal acquisition covered the entire heart and great vessels. Other parameters: FOV 250x250mm, voxel size $2.5mm^3$ isotropic, TE 2.8ms, TR 4.1ms, flip angle 15°, and 16-23 phases per cardiac cycle depending on participant heart rate, scanner and practical acquisition time [25]. Data quality was assessed visually as a lack of image artefact (in the form of phase encoded ghosting from poor cardiac gating or respiratory motion) by an experienced observer.

2D-PC acquisitions were performed at three positions: the main pulmonary artery (MPA), AscAo and aortic valve levels (AV).

Positioning for these locations was performed during the 4D-flow acquisition as a time-saving measure using the MRA volume. Additional refinement of position was performed using cine data as necessary, and repeat views were obtained when the acquisition was deemed inaccurate by the operator. Ongoing training and feedback on this process was performed routinely by the clinical reporting team. Other parameters: FOV 300x300mm, in-plane resolution 1.4mm^2, TE 3.3ms, TR 5.5ms, flip angle 20°, VENC 250cm/s, slice thickness 8mm resulting in 18 to 20 contiguous slices from base to apex, and 25 to 30 phases covering an entire cardiac cycle depending on heart rate.

SAX cine data was acquired using a steady-state, free-precession (SSFP) sequence (FOV 350x350mm, in-plane resolution 0.74mm^2, TE 1.5 ms, TR 3.4 ms,; flip angle: 45°). Images were acquired in a standard short-axis orientation with a slice thickness of 8 mm for 25 to 30 phases covering a complete cardiac cycle. The entire heart including the left and right ventricles and atria was covered with contiguous slices from base to apex resulting in 18 to 20 slices.

MRA was acquired using a single end-expiratory breath-hold of 15-17sec. Parameters: in-plane resolution 1.0mm^2, slice thickness 4mm, TE 1.3ms, TR 4.8ms, flip angle 30°.

PS-DGE sequences were acquired using the following parameters: in-plane resolution 1.0mm^2, slice thickness 8mm, TE 1.8ms, TR 4.0ms, flip angle 25°. A mid-ventricular short-axis Look-Locker acquisition was obtained to select an appropriate TI, followed by long-axis DGE series. Additional "crosscuts" or repeat acquisitions with different TI were also acquired at operator discretion.

Additional long-axis cine acquisitions were performed in multiple standard planes (LVLA, 4CH, LVOT, RVOT, RVLA) using the same number of time steps and in-plane resolution as the SAX views.

Table S1. Detailed Summary of Acquisition Protocol.

Scan type	Purpose/Cardiac Measure	Scan Protocol	Time	Total
	CMR_{FAST}			
scout	Planning - SB 3D-cine, MRA & 4D-flow		1min	1min
SB 3D-cine	Volumes & EF	2mm isotropic	1min	2min
MRA	Aortic and pulmonary dimensions	1mm isotropic	1min	3min
4D-flow	Aortic flow, MPA flow	VENC 170	9min	12min
Look-Locker	Aortic flow, MPA flow	To check TI time	1min	13min
SAX DGE	Exclude DGE	8mm, gap 4mm	5min	18min
LVLA/4CH DGE	Exclude DGE	8mm	3min	20min
	CMR_{STD}			
Additional DGE	As deemed necessary by Radiographer	6 slices along 3 principal axes.	2 min	22min
2D-PC Ao	Aortic flow	VENC 250	1min	23min
2D-PC MPA	MPA flow	VENC 250	1min	24min
2D-PC valve	Aortic and pulmonary valve dimensions	VENC 250 unless AS	1min	25min
SAX cine	Volumes & EF	8mm	8min	33min
Long-axis cine	4CH, LVLA, RVLA, LVOT, RVOT	To check TI time	5min	38min
SAX DGE	Exclude DGE	8mm, gap 4mm	4min	42min
LVLA/4CH DGE	Exclude DGE,	8mm	3min	45min
	Additional as indicated			

4D-flow	40 VENC		30sec
2D-PC	30 VENC MV	8mm	2min

Abbreviations: 4CH, four-chamber; 2D, two-dimensional; 3D, three-dimensional; 4D, four-dimensional; Ao, aorta; AS, aortic stenosis; DGE, delayed gadolinium enhancement; EF, ejection fraction; LA, left atrium; LV, left ventricle; MPA, main pulmonary artery; MRA, magnetic resonance aortogram; OT, outflow tract; RV, right ventricle; SAX, short-axis; SB, single breath-hold; TI: inversion time; VENC, velocity encoding.

S2. CMR Reporting and Image Analysis Environment

Figure S1 summarises the data flow for clinical reporting and research analysis. SAX cine and 2D-PC were analysed using Circle Cardiovascular Imaging (CVI42 v5.12.1, Vancouver, Canada), a leading cardiovascular post-processing software remotely deployed via a central server (iCoreLab). Datasets of 3D-cine and 4D-flow were analysed in their true 3D views without reformatting to 2D slices in Arterys (Tempus Radiology v31.0.0, San Francisco, CA, USA), a web-based software application which facilitates clinical data visualisation and semi-automated flow and volume analysis. Clinical reporting also used standard Picture Archiving and Communication System (PACS) viewers for extra-cardiac review. Typical 2D-PC and 4D-flow analysis views are provided in Supplemental Materials Figures S2 and S3.

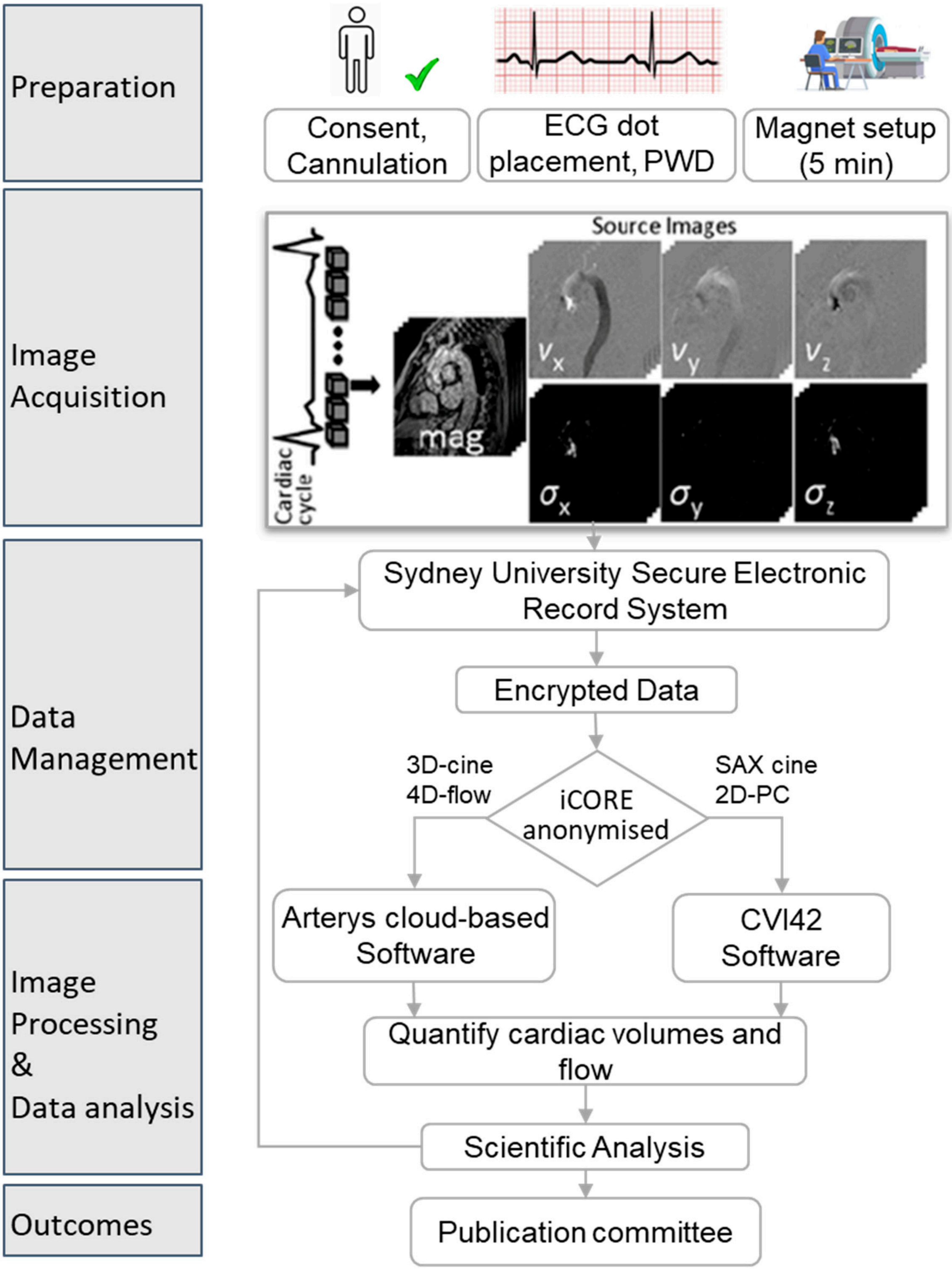

Figure S1. Data flow process for clinical reporting and research analysis. PWD: pulsed wave doppler; SAX: short-axis.

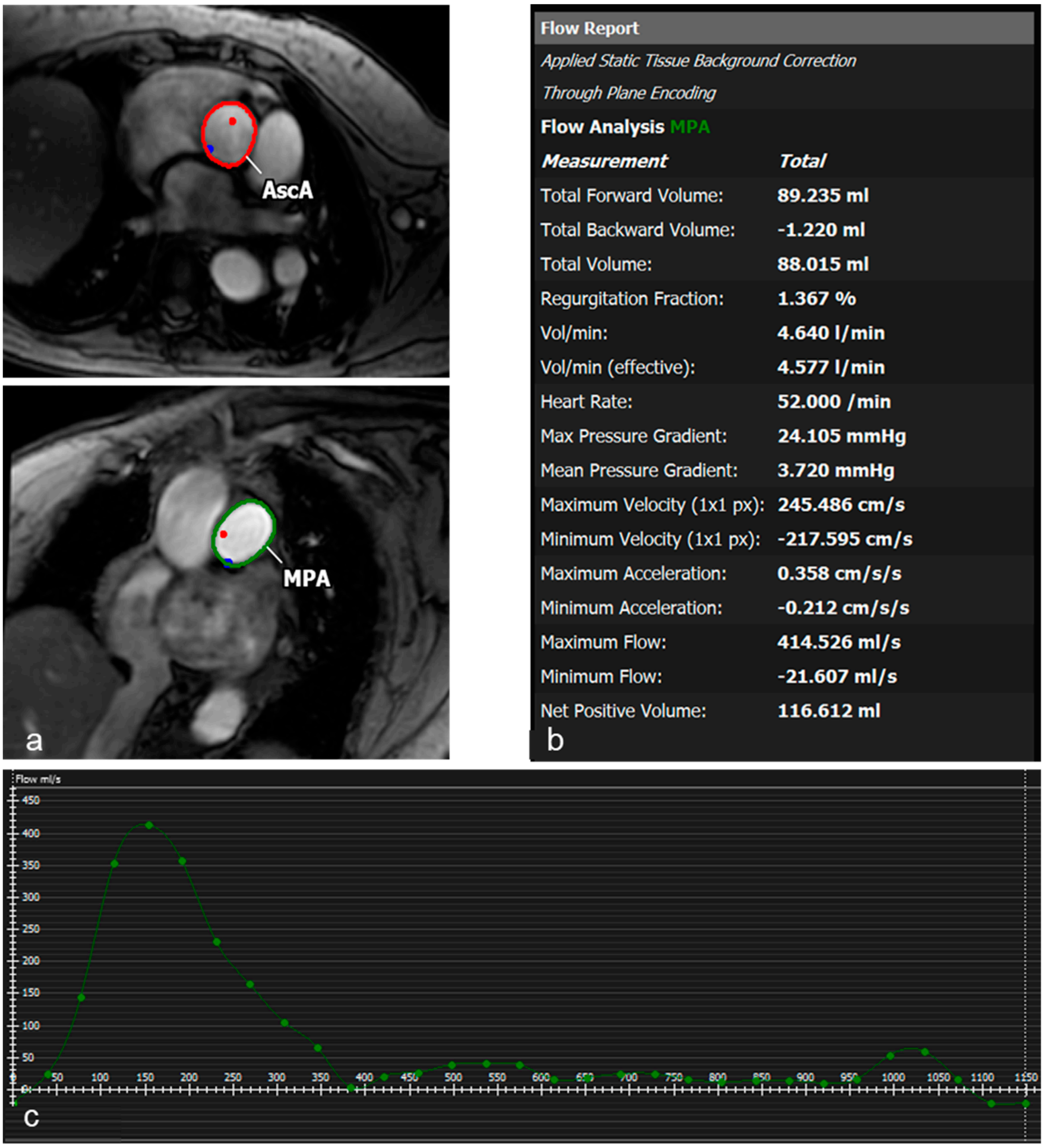

Figure S2. Typical 2D-PC contouring in CVI42. a) contouring of regional of interest (ROI); b) numerical outputs calculated from ROIs; c) flow curve calculated from ROIs. AscA: ascending aorta; MPA: main pulmonary artery.

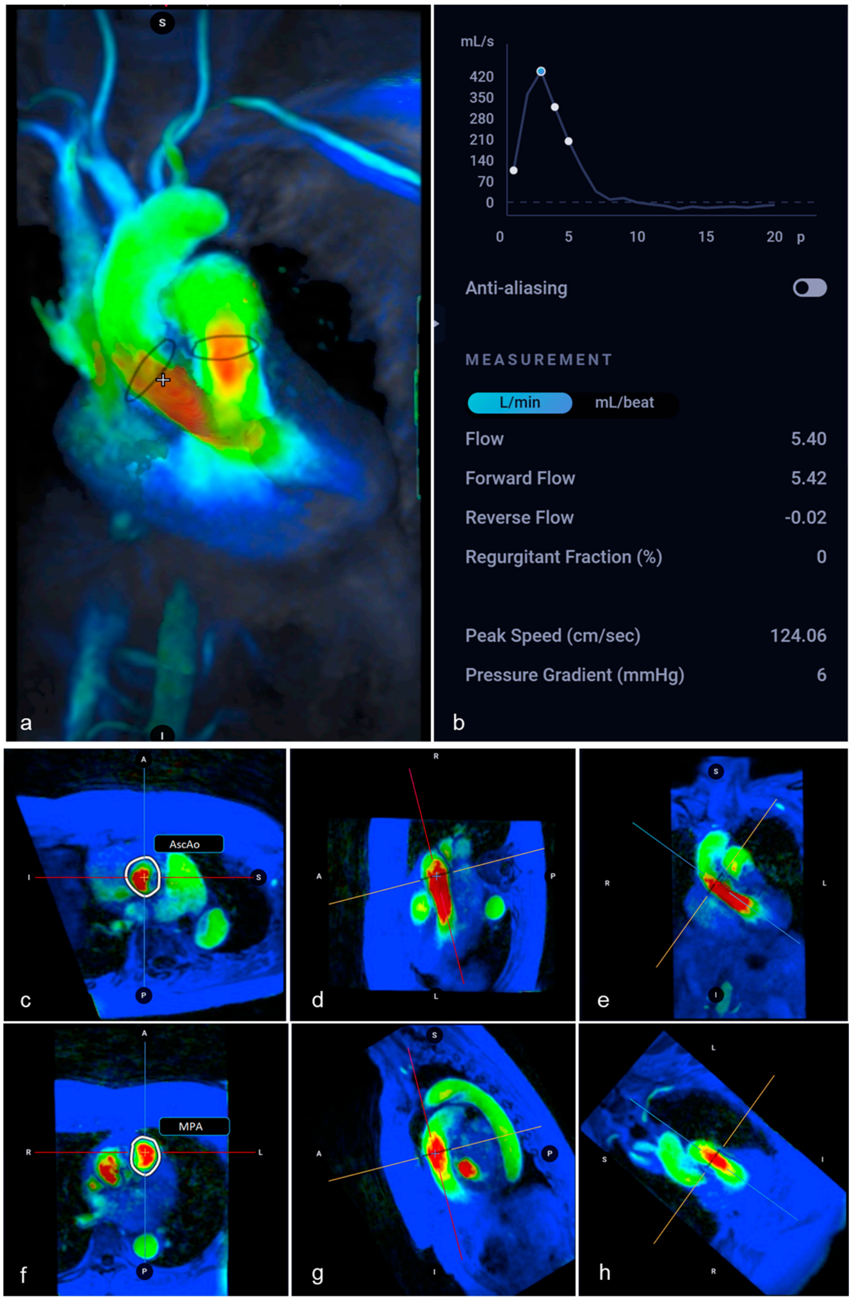

Figure S3. Typical 4D-flow contouring in Arterys. a) whole-heart visualisation for orientation; b) numeric outputs; c-h) 3-plane contouring views for the ascending aorta (AscAo) (c-e) and pulmonary artery (MPA) (f-h) with axial (e&f), sagittal (d&g) and coronal (e&h) views.

S3. Specific image analysis details

S3.1. Specific Analysis Details - 2D-PC

Acquisition and analysis of 2D-PC data typically requires expert CMR experience to produce accurate results [35]. Current state-of-the-art eddy current correction has improved considerably over the past decade. Historically correction for ‘baseline’ flow was a major limiter of accuracy and reproducibility, often influenced inconsistently by measurer’s clinical experience, especially when diastolic flow was artefactually affected by phase offsets due to incomplete eddy current correction. In view of the improved eddy current correction employed

in current software such as CVI42, the standard correction applied within the package was utilised during image analysis without further user input.

Contouring was performed using the following approach (Figure S2):

1. SAX stack images were inspected over the cardiac cycle to evaluate for image quality, completeness and artefact. If the dataset was incomplete or subject to significant artefact, the study was tagged for expert review. Suboptimal image quality or the presence of artefact only precluded the study from contouring if the data was deemed uninterpretable.
2. Peak systole was identified from bulk flow patterns. AscAo and MPA vessel lumens were contoured in peak systole by manually placing a closed curve around the region of interest (ROI), taking care to trace the boundaries near the vessel edge to eliminate interference from nearby vessels and to exclude excessive inclusion of static tissues that will result in integration error (Casciaro et al. 2021). The annotators were trained to maximise phase contrast between blood and static tissue by adjusting windowing settings to ensure correct identification of lumen boundaries.
3. The ROI was automatically propagated across all phases of the cardiac cycle. These phases were visually inspected and ROIs manually corrected if necessary. The measurements were automatically extracted and saved to a central database, and the contours exported to a structured file system for future analysis.

S3.2. Specific Analysis Details - 4D-Flow

4D-flow acquisition is volumetric and requires minimal planning and placement of acquisition volume at the time of scanning. However, the determination of appropriate analysis locations within the 3D volume during image analysis becomes paramount in order to obtain reproducible and precise flow measurements [36]. Annotators underwent specific training to identify the anatomical analysis locations to make flow measurements in a standardised, repeatable manner. Similar to 2D-PC contouring, eddy current correction was performed using the automatic algorithm in Arterys without further user input.

Contouring was performed using the following approach (Figure S3):

1. 4D-flow data were visualised in Arterys in a 3-plane view (SAX, sagittal and coronal views) and inspected over the cardiac cycle to assess image quality and completeness. The study was tagged for expert review if considered incomplete or affected by artefact. Suboptimal image quality or the presence of artefact only precluded the study from contouring if the data was deemed uninterpretable.
2. Determination of analysis locations was performed in peak systole using standardised anatomical landmarks via codified steps on magnitude images overlaid with flow velocity colour maps. Annotators navigated to the locations using a standard 3D orthogonal view within Arterys.
    a. For AscAo, in coronal view, the image was oriented such that the cross-sectional ROI plane was perpendicular to the aortic

vessel wall. The ROI was then moved from the AV superiorly until the image plane transected the right pulmonary artery (RPA), then the plane of the ROI readjusted to be perpendicular to the vessel wall.

   b. For MPA, the ROI was placed 4cm distal to the pulmonary valve, approximately midway between the pulmonary valve (PV) and the pulmonary bifurcation, centred within the MPA lumen. The plane of the ROI was then adjusted to be perpendicular to the vessel wall.

3. Once the analysis locations were determined for AscAo and MPA, the ROIs were contoured in SAX by placing a closed curve manually around the vessel lumen in peak systole, taking care to exclude flow from adjacent structures. The contour was then propagated automatically to all phases which were visually inspected and manually corrected as required.
